# Real-Time Dense Reconstruction with Binocular Endoscopy Based on StereoNet and ORB-SLAM

**DOI:** 10.3390/s23042074

**Published:** 2023-02-12

**Authors:** Jiayi Huo, Changjiang Zhou, Bo Yuan, Qing Yang, Liqiang Wang

**Affiliations:** 1 State Key Laboratory of Modern Optical Instrumentation, College of Optical Science and Engineering, Zhejiang University, Hangzhou 310027, China; 2 Research Center for Humanoid Sensing, Zhejiang Lab, Hangzhou 311100, China

**Keywords:** endoscope, binocular matching, 3D stitching, SLAM, deep learning

## Abstract

Binocular endoscopy is gradually becoming the future of minimally invasive surgery (MIS) thanks to the development of stereo vision. However, some problems still exist, such as the low reconstruction accuracy, small surgical field, and low computational efficiency. To solve these problems, we designed a framework for real-time dense reconstruction in binocular endoscopy scenes. First, we obtained the initial disparity map using an SGBM algorithm and proposed the disparity confidence map as a dataset to provide StereoNet training. Then, based on the depth map predicted by StereoNet, the corresponding left image of each depth map was input into the Oriented Fast and Brief-Simultaneous Localization and Mapping (ORB-SLAM) framework using an RGB-D camera to realize the real-time dense reconstruction of the binocular endoscopy scene. The proposed algorithm was verified in the stomach phantom and a real pig stomach. Compared with the ground truth, the proposed algorithm’s RMSE is 1.620 mm, and the number of effective points in the point cloud is 834,650, which is a significant improvement in the mapping ability compared with binocular SLAM and ensures the real-time performance of the algorithm while performing dense reconstruction. The effectiveness of the proposed algorithm is verified.

## 1. Introduction

MIS has made great progress in the past decade. With the development of stereovision technology, 3D endoscopy has been increasingly used due to its rich depth of information [1]. How to accurately reconstruct 3D information and splice the obtained point cloud with the help of endoscopy has also become a research hotspot. This study was started from previous research on monocular endoscopy and binocular endoscopy, which are widely used methods at present. Image depth estimation is the basic module of SLAM. Widya et al. used Shape from Motion (SfM) to achieve complete stomach reconstruction based on monocular endoscopy, but the reconstruction results were sparse and lacked physical scale information [2]. With the development of deep learning, monocular depth prediction has gradually become the focus of research [3]. Li and Liu et al. realized the super resolution of the predicted depth map by using the color image super resolution model, which greatly affected the accuracy [4,5]. In order to obtain a more accurate depth map, a deeper deep learning network was built to achieve a more accurate depth estimation, but it is greatly affected in terms of efficiency [6,7,8]. Binocular endoscopy benefits from the existence of disparity information. Depth information is easier to obtain under light sources such as white light, xenon lamps, and lasers [9,10]. The SGBM algorithm proposed by Hirschmuller et al. has been widely used in traditional binocular scenes due to its advantages of fast speed and high precision [11]. Xia et al. improved the SGBM algorithm to improve its accuracy of reconstruction in the contexts of sparse textures and a large number of pathological regions in the endoscope scene [12]. With the development of neural networks, three-dimensional matching technology based on deep learning has been applied more widely. However, there is no publicly available binocular dataset in the field of MIS. To overcome this problem, researchers have adopted traditional scene datasets [13], self-supervised convolutional neural networks [14], and simulation models rendered using Blender [15,16], etc., but the results differ from those of traditional scenes.

SLAM, a wide-range splicing framework widely used in the field of stereo vision, is also gradually becoming favored by researchers in the field of medical imaging. Among SLAM frameworks, the ORB-SLAM framework proposed and improved by Mur-Artal has attracted much attention due to its excellent computational efficiency [17,18]. In the medical field, monocular endoscopy is still widely used. Wang et al. used a monocular bronchoscope and ORB-SLAM to achieve feature point model reconstruction of the bronchus, but the results were very sparse, only restoring the general shape of the bronchus [19]. Mahmoud et al. used monocular endoscopy combined with ORB-SLAM to estimate the position and trajectory of the endoscope and conducted semi-dense, three-dimensional reconstruction, which showed low efficiency and inaccurate physical scale information [20]. Compared with monocular cameras, binocular and RGB-D cameras can simultaneously acquire the texture and physical scale of objects. In complex surgical environments, binocular cameras and RGB-D cameras have greater robustness and better results in terms of computational efficiency and density. Whelan et al. implemented ElasticFusion for dense 3D panoramic reconstruction using an RGB-D camera [21], but due to the size and power consumption of the RGB-D camera [22], it could not be applied to the endoscope scene. Therefore, Docea used a binocular camera to combine ElasticFusion with ORB-SLAM and verified it using a laparoscope so as to obtain a more accurate trajectory and denser point cloud [23]. It can be seen that the binocular camera has a good prospects in dense stitching.

The proposed algorithm in this study for the dense reconstruction of binocular endoscopy is based on deep learning and its process is as follows: (1) An initial disparity map is obtained via the traditional SGBM method, and the holes in the disparity are filled according to the basic continuous characteristics of the disparity in the MIS scene. The disparity confidence map is introduced in the dataset. (2) The depth map sequence is obtained by real-time matching and reconstructing the left and right image sequences of the binocular endoscope through StereoNet. (3) The ORB-SLAM algorithm in RGB-D mode is used as the baseline in the left view, and the binocular reconstruction result predicted by StereoNet is used as the depth map of the RGB-D camera. The left view provides RGB information, which overcomes the limitation that the traditional binocular SLAM cannot achieve dense reconstruction, and still retains the physical scale information while ensuring the calculation speed. The realization of real-time dense reconstruction in the endoscopic scene provides doctors with richer information in the surgical scene, improves the depth perception ability of doctors, helps to reduce the risk of surgery, improves the prognosis of patients, and reduces the training cycle of doctors, which lays a foundation for the development of surgical navigation.

## 2. Methods

### 2.1. Framework

**Figure 1:**
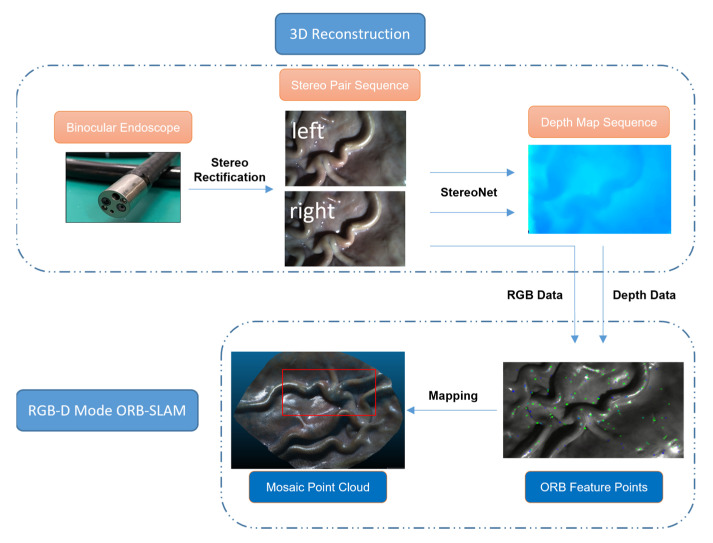
Framework of our method.

The overall framework of our algorithm is shown in Figure 1. It is different from the traditional binocular SLAM process. We first used Zhang’s calibration method to calibrate the images acquired through the binocular endoscope to obtain the stereo rectification parameters of the endoscope. However, the calibrated images are not directly input into the SLAM system for binocular reconstruction and mapping. Instead, the image sequences are composed and input into the pre-trained StereoNet, and the trained StereoNet can process the image pair sequence of the endoscope to obtain the corresponding depth map sequence. Then, taking the left image in each image sequence as the reference, the RGB-D mode in the ORB-SLAM algorithm is used to input the left image sequence as RGB information and the corresponding depth map sequence, calculate the point cloud, and find the feature points to realize the real-time dense reconstruction of the endoscopic scene. The large-scale mosaic of the scene is completed according to the image sequence to realize the mapping of the entire endoscopic scene.

### 2.2. Dataset and StereoNet

#### 2.2.1. Dataset

**Figure 2:**
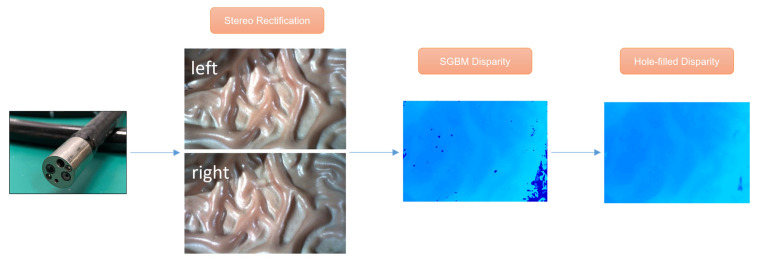
Procedure of proceeding dataset.

Since there is no public clinical or rendering dataset of binocular endoscopy, we propose a fast and simple method for binocular endoscopy image datasets under the gastrointestinal tract, which can generate many datasets in a short time for the training of deep learning neural networks. The production flow is shown in Figure 2, where the SGBM algorithm, which is widely used for binocular image reconstruction, is first applied to obtain the initial disparity map. However, due to the ill-conditioned areas such as over-exposure, occlusion, and noise in the gastroscopy images, the robustness of the algorithm is poor and many disparity holes and some mismatching occur. Aiming at the hole in the process of ill-conditioned region matching, we propose a hole-filling algorithm, which takes advantage of the characteristic that the surface depth of most organs in the scene we photographed often vary continuously. Therefore, the corresponding disparity value is also continuously changed, and iterative mean filtering is performed on the hole’s boundary points to replace the original error value by the average of the effective values in the neighborhood of the hole boundary pixels, thus filling the hole. In the hole detection step, the Canny detection operator is used to detect and extract the initial disparity map edge, and it is filled with morphology information. The pixels on the hole boundary of the first detected edge region are processed by mean filtering, and the disparity map is updated. Then the process is repeated until the holes in the disparity map cannot be detected by the Canny edge detection. Median filtering is performed on all pixels in the disparity map that are below the set threshold to remove the residual noise and complete the process of filling the holes.

Because of the problems of the SGBM algorithm, the image after hole-filling still has a lot of noise caused by mismatching, so the result is not completely reliable. Therefore, the disparity confidence map (DCM) is introduced as the evaluation index of its reliability. This method adopts the idea of backward extrapolation. Since the disparity map is obtained according to the left and right views, the right view can be reconstructed by the left disparity map and the left view without the influence of noise, and the right view can be reconstructed similarly. However, the existence of an error disparity value will lead to differences occurring between the intensity of the reconstructed image and the original image, which reflects the degree of the disparity value’s error at this point. The larger the difference, the greater the deviation and the lower the reliability. Therefore, the calculation method of DCM is as follows: First, the difference between the reconstructed view and the original view is calculated. Let $I_{l}$ and $I_{r}$ be the input left and right images, respectively, and disp be the input left disparity map. Then the formula for calculating the difference diff between the reconstructed right image and the input right image in a fixed size window is as follows:

(1) $$diff\left(i,j,c\right)=\frac{1}{N}\overset{i+\frac{k}{2}}{\underset{x=i-\frac{k}{2}}{\sum }}\overset{i+\frac{k}{2}}{\underset{y=i-\frac{k}{2}}{\sum }}\left|\frac{I_{l}\left(x,y,c\right)}{\bar{I_{l}\_block}}-\frac{I_{r}\left(x-Disp\left(i,j\right),y,c\right)}{\bar{I_{r}\_block}}\right|$$

where i and j represent the row and column of the input image, respectively, c represents the channel of the image, k is the size of the support window to calculate the diff of each pixel, and $\bar{I_{l}\_block},\;\bar{I_{r}\_block}$ are the average values of the left and right images over the support window of k×k, respectively. After that, the final diff is obtained by averaging the difference values of each channel, as follows:

(2) $$Diff\left(i,j\right)=\frac{1}{3}\overset{3}{\underset{c=1}{\sum }}diff\left(i,j,c\right)$$

Finally, the disparity map is normalized in the same way as the linear function is normalized, and a value of 1 is added to the negative number to obtain the normalized disparity confidence map:

(3) $$DCM=1-\left|\left|Diff\right|\right|$$

The closer the value of DCM is to 1, the more accurate the disparity value and the higher the confidence. At the same time, considering the difference in the absolute difference between the left and right views of the same viewpoint in the real scene, the pixel value information in the support window of size $\left(k+1\right)\times \left(k+1\right)$ centered at the point $\left(i,j\right)$ is used to calculate the confidence $DCM\left(i,j\right)$ of $disp\left(i,j\right)$. In this way, a more evenly distributed result can be obtained, and in the training of the proposed algorithm, we adopt a support window of 5×5. Combining the disparity map after hole-filling with DCM, we obtain the dataset in the binocular endoscope scene used to train our StereoNet.

#### 2.2.2. StereoNet

**Figure 3:**
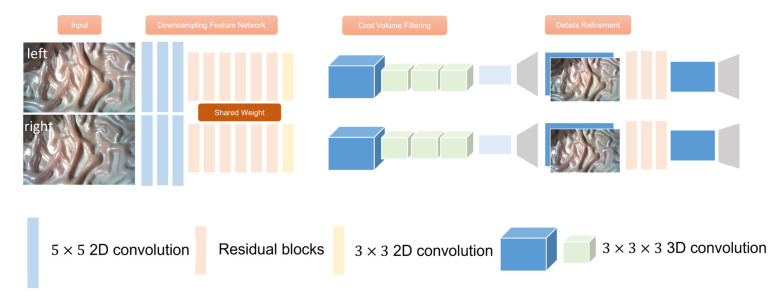
Network Structure of StereoNet.

Our end-to-end disparity estimation network is mainly based on StereoNet proposed by [24] in 2018, and all the steps of traditional binocular disparity calculation such as cost matching, cost aggregation, disparity calculation, and optimization are implemented by convolutional networks. The network structure is shown in Figure 3. It consists of feature extraction network, cost calculation and filtering network, disparity calculation, and layer-by-layer optimization network. StereoNet uses a low-resolution feature map and an edge-aware up-sampling module to achieve high-precision real-time disparity prediction. StereoNet performs well in weakly textured regions while being computationally efficient.

StereoNet uses a Siamese network to down-sample the left and right images of the input, uses three 5×5 convolution layers and a step size of 2 to down-sample the input image. It then extracts image features through six residual blocks consisting of 3×3 convolution, batch regularization, and the ReLU function. The 1/8 resolution feature representation of the image is obtained. In the cost aggregation module, the difference between the features of the left and right graphs is used as the initial cost amount, and then through four 3D convolution layers consisting of 3×3×3 convolution, batch regularization, and the ReLU activation function, the cost amount with a resolution of length×width×disparity is obtained by filtering. Finally, the optimal disparity value corresponding to each pixel is regressed from the cost quantity by the differentiable soft argmin. When training, we cannot obtain the truth value, so we introduce DCM as a criterion to evaluate the truth value we create, and introduce DCM into our loss function, the corresponding loss function will be modified as follows:

(4) $$loss=\frac{1}{H}\frac{1}{W}\overset{H}{\underset{i=1}{\sum }}\overset{W}{\underset{j=1}{\sum }}DCM\left(i,j\right)\left|d\left(i,j\right)-d{\left(i,j\right)}^{’}\right|$$

where H and  W represent length and width, respectively, d is the predicted disparity value, and d’ is the ground truth value.

### 2.3. SLAM-Based Real-Time Dense Reconstruction

We use the ORB-SLAM framework for binocular endoscopic camera tracking and stitching, which has obvious advantages compared to other SLAM frameworks. The ORB-SLAM framework adopts the feature point matching method and uses ORB feature points as landmarks in the SLAM framework. The ORB feature improves the non-directional problem of the FAST detector by calculating the main direction of feature points, and uses the extremely fast binary descriptor BRIEF, which is combined with the direction information calculated before, so that the speed of image feature extraction is greatly improved. At the same time, the image pyramid is used to detect corner points on each level of the pyramid to achieve scale invariance. Thanks to the fast speed and scale invariance of ORB feature points, it is more suitable for application in endoscopic scenes. At the same time, in order to improve the robustness of the whole system, and considering that the image features in the gastrointestinal tract are not very obvious, we set the ORB to extract a maximum of 2000 feature points. This allows for better performance in the weak texture scene of gastrointestinal endoscopy.

After the conventional binocular SLAM is calibrated, the feature points in the left and right images are extracted, and the coordinates of the feature points are set to $\left(x_{L},y_{L},x_{R}\right)$, where $\left(x_{L},y_{L}\right)\;$ are the corresponding coordinates of the feature points in the left image, and $x_{R}$ is the horizontal coordinate of the point matching the left image in the right image. Because the epipolar line of the binocular camera has been calibrated, the horizontal coordinates of the feature points in the right image can be easily obtained. The binocular mode of ORB-SLAM is based on feature points and key frames. It matches the feature points between the two frames to achieve sparse feature point splicing results, which cannot achieve dense reconstruction. In the ORB-SLAM in RGB-D mode, since the RGB-D camera provides a color map and a depth map, the RGB-D camera’s data are transformed into a point cloud by estimating the position of the camera, and the depth information is mapped to the texture information. Finally, the point clouds obtained from multiple stereo image pairs are stitched to obtain a point cloud map composed of discrete points.

In our algorithm’s framework, the left image information of the binocular endoscope is used as the color information in the simulated RGB-D camera, and the binocular depth map predicted by StereoNet is used as the depth information in the simulated RGB-D camera. ORB-SLAM extracts the feature points in the left image as landmarks in the SLAM framework, and estimates the camera pose. Thus, the point clouds corresponding to each left image and depth map are generated and spliced to obtain the final point cloud map of dense reconstruction. With the help of binocular accuracy, our algorithm avoids the inefficiency and low accuracy of monocular depth prediction, and successfully replaces the RGB-D camera in the endoscopic environment, showing improvement in many aspects such as accuracy, density, and real-time performance.

## 3. Results

In this article, a real-time reconstruction system based on binocular endoscopy was built, mainly composed of a high power LED light source, a binocular endoscope mirror body and signal processing circuit, and image processing workstations. The system’s parameters acquired by Zhang’s calibration method are as follows: the focal length is 1059.6 pixels, the baseline length is 5.9 mm, the CMOS size is 1.75 × 1.75 μm, and the camera principal point positions $c_{x}$ and $c_{y}$ are 633.6 and 367.1 pixels, respectively. Through this system, pictures of a pig stomach purchased from a supermarket were collected and input into the workstation for processing. The data acquisition of the true value is realized by the 3D scanner Einscan-pro-2x-plus from Hangzhou SHINING 3D Technology, the accuracy of the scanner can reach 0.04 mm. The 3D point cloud of the pig stomach obtained by the scanner can be used as the true value data for data analysis and processing.

In our experiments, a computer with an Intel Core i9-9900K CPU, NVIDIA GeForce RTX 2080Ti graphics card and 32 GB memory was used for StereoNet training. According to the proposed binocular data set generation method, 2800 pairs of binocular images of the stomach model and the corresponding disparity truth values were generated. We used 2500 pairs for the training set, 200 pairs for the validation set, and the remaining 100 pairs for the test set. After several rounds of parameter tuning, we obtained the trained model parameters that are most suitable for the characteristics of the endoscopic scene. In the training, the RMSprop optimizer was used to optimize and update the network parameters. The image of the training set was randomly clipped to the size of 384×768 and input into the network. The batch size of the training set was set to 24, the batch size of the verification set to 4, and the learning rate was set to $10^{-3}$. The maximum disparity value was set to 168, and the training rounds were set to 100.The trained algorithm and the subsequent ORB-SLAM algorithm were implemented on a Ubuntu 16.04 system, where StereoNet is implemented by the PyTorch 1.9.1 deep learning framework. The ORB-SLAM system is implemented on C++ without GPU acceleration. In terms of time consumption, the traditional SGBM algorithm takes 365 ms to process a pair of stereo images with size of 1280×720. Because of the iterative process of detection and filling, the average time of the method of hole-filling is 7700 ms for different stereo images. The two methods basically cannot meet the needs of real-time reconstruction. However, StereoNet only takes 35 ms to predict a depth map, which is much more efficient than the previous two methods. In terms of the stitching time of ORB-SLAM, the stitching time between every two frames is about 15 ms. Using StereoNet for prediction, the depth prediction and stitching can be completed in about 50 ms, and the frame rate can reach 20FPS, which basically reaches real-time performance.

**Figure 4:**
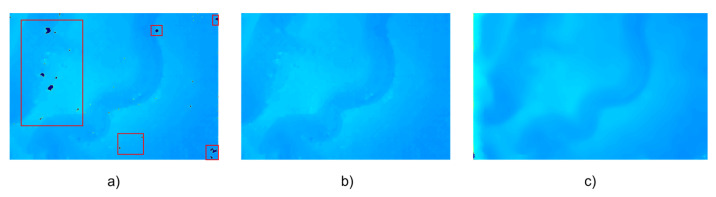
(**a**) Disparity map by SGBM. (**b**) Hole-filled Disparity map by SGBM. (**c**) Disparity map by StereoNet.

A binocular endoscope was used to acquire left and right images, SGBM and SGBM with hole-filling and StereoNet prediction, respectively, were adopted to process the acquired images and obtain the depth map of a single image. The results are shown in Figure 4. There are many holes in the depth map obtained by the traditional SGBM method, as shown in the red rectangles in Figure 4a. After hole-filling, the results obtained by the SGBM algorithm are smoother and the noise is reduced. The results predicted by StereoNet will not only fill the holes in the traditional algorithm, but the resulting image will also be smoother, in line with the characteristics of the continuous depth of human organs.

**Figure 5:**
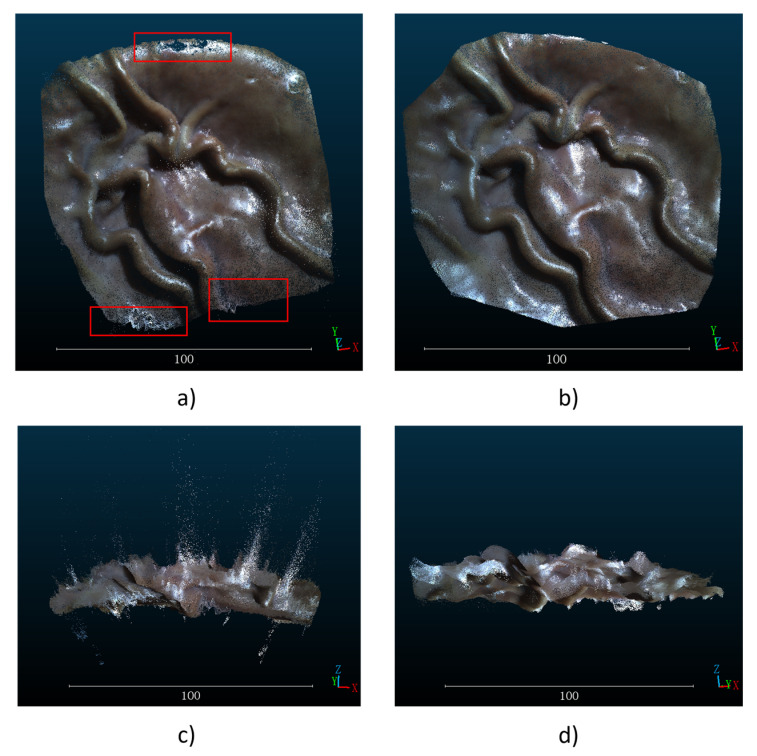
Different views of the depth prediction method. (**a**) Top view of mosaicked point cloud map by SGBM. (**b**) Top view of mosaicked point cloud map by StereoNet. (**c**) Side view of mosaicked point cloud map by SGBM. (**d**) Side view of mosaicked point cloud map by StereoNet.

Since the hole-filling method takes too long to reconstruct a single frame, only the images obtained by the traditional SGBM algorithm StereoNet are used for mosaicking when using ORB-SLAM. After the multiple depth map sequences are obtained by SGBM and StereoNet and the image sequences taken by the left camera are used as the input of ORB-SLAM in RGB-D mode, a wide-range mosaicked point cloud image is obtained as shown in Figure 5. In Figure 5, the point cloud images obtained by the two methods are compared from the top view and side view. It can be seen that after converting the depth maps into point cloud images, there are a large number of outliers in the depth maps obtained by the traditional SGBM methods in Figure 5a,c. Moreover, unfilled holes are shown in the red rectangles in Figure 5a and the shortcoming of not being smooth enough affect the final stitching results. In Figure 5b,d, compared with the result by traditional SGBM algorithm, the depth map predicted by StereoNet not only realized the filling of holes, but also had fewer noise points. In the mosaicked point cloud map, there were fewer outliers in the whole pig stomach. The smooth result of StereoNet’s prediction could be observed from different perspectives, and the reconstructed whole pig stomach was closer to the ground truth.

**Figure 6:**
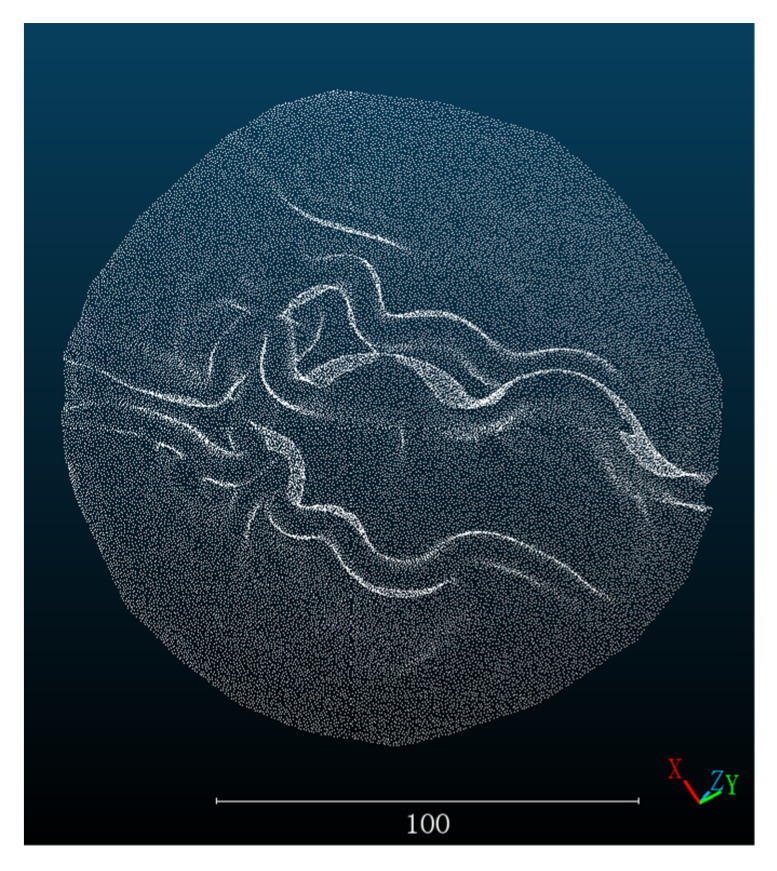
Ground truth value scanned by 3D Scanner.

**Figure 7:**
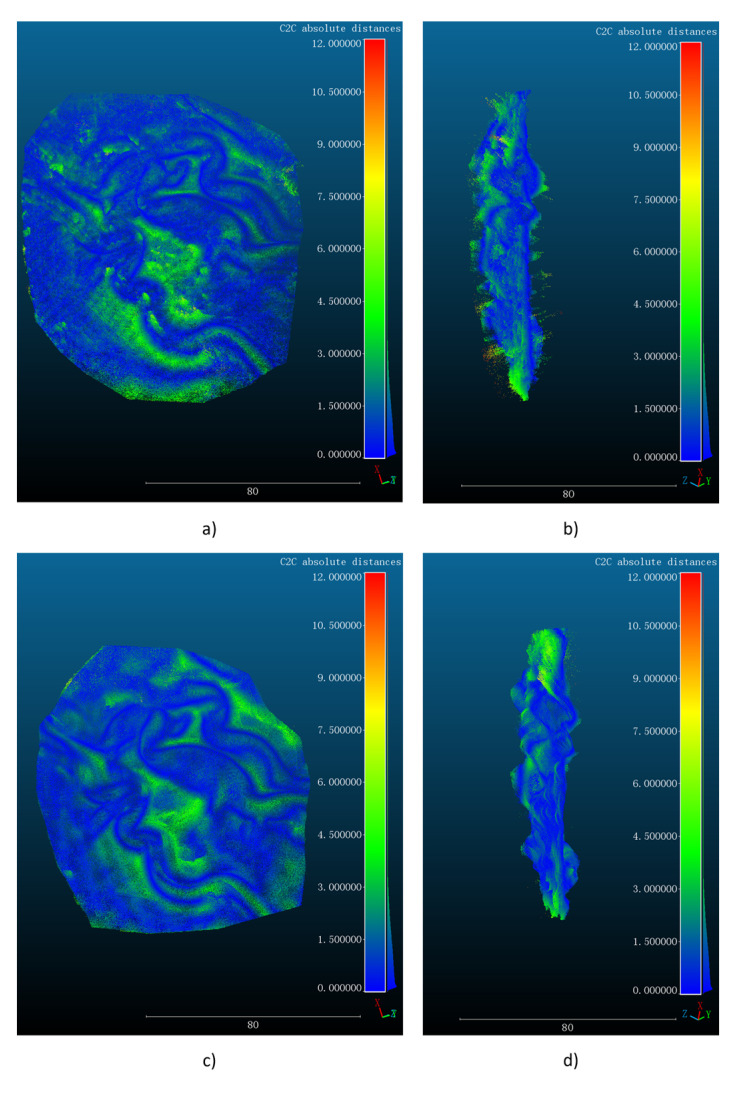
(**a**) Top view of cloud map registered by SGBM. (**b**) Side view of cloud map registered by SGBM. (**c**) Top view of cloud map registered by StereoNet. (**d**) Side view of cloud map registered by StereoNet.

After that, we used the pig stomach point cloud obtained by the 3D scanner as the ground truth. In order to facilitate information processing, increase the computing speed, the point cloud image obtained by the 3D scanner was preprocessed by segmentation and cutting as shown in Figure 6. Moreover, the point cloud image obtained by three methods is registered with the truth value. We use the built-in point cloud registration method in the software CloudCompare. Firstly, the corresponding feature points are manually selected for coarse registration, then the integrated ICP algorithm is used for fine registration, and finally the RMSE of registration is calculated. The RMSE of the registration results is used to evaluate the error of the three methods. The RMSE is calculated as follows.:

(5) $$RMSE=\sqrt{\frac{1}{n}\overset{n}{\underset{i=1}{\sum }}{\left(do_{i}-ds_{i}\right)}^{2}}$$

where $do_{i}\;$ and $ds_{i}$ are the depth values of the reconstructed and scanned points respectively, and n is the number of reconstructed point clouds. We evaluate the mosaicked point clouds obtained by the traditional SGBM algorithm and StereoNet, and the evaluation results are shown in Figure 7 in different views. In particular, the RMSE of registration between the SGBM and scanned point cloud is 2.134 mm, while the RMSE of registration between the StereoNet prediction and the scanned point cloud is 1.260 mm, which is significantly better than that of the point cloud obtained from SGBM reconstruction.

In terms of point cloud density, the proposed dense reconstruction method not only completely retains the physical scale information of the target organs and completes their dense reconstruction with high precision, but it also obtains the point cloud information of 834,650 points through the stitching of 160 frames of images and depth sequences. It provides sufficient support for the subsequent mapping of surgical navigation.

## 4. Discussion

In this article, a new algorithm framework for real-time dense reconstruction is proposed. Combined with a binocular endoscope, this system can realize the dense reconstruction of MIS scenes. Experiments were carried out on simulated stomach models and real pig stomachs, and accuracy at the millimeter level was reached. In the aspect of dataset construction of the deep learning model, we innovatively proposed a set of simple production methods. With the help of a binocular endoscope, depth maps and DCMs  obtained by the SGBM algorithm were used to complete the construction of the dataset, which provided the direction for the construction of the binocular depth prediction dataset. Using binocular equipment, datasets of different scenarios can be obtained, so there is no need to use traditional datasets for a variety of environmental training scenarios, which improves robustness and accuracy. In terms of depth prediction, data from the binocular endoscope are used. Compared with monocular endoscope prediction, binocular endoscope prediction can provide more abundant information and has a higher computational efficiency. Accurate binocular matching and depth prediction can be achieved without the use of a deep neural network, which can meet the needs of the large-scale reconstruction of clinical organs. At the same time, the results obtained by using the deep learning method to achieve stereo matching of binocular endoscope data are more accurate. Compared with the results obtained by the traditional SGBM algorithm, the results are not only smoother and less noisy, but they also fill the holes in the SGBM algorithm.

In terms of density, compared with the results obtained by the traditional SLAM algorithm [19] and SfM algorithm [2], we have achieved a denser point cloud reconstruction on the basis of restoring the basic shape of the stomach. This method not only allows surgeons to observe the basic shape of the organ before surgery, but it can also be used in surgical navigation during surgery, providing surgeons with more abundant information. At the same time, the physical scale information of the organ is fully retained, which is of great significance for the measurement of the size of potential lesions in clinic settings.

Our method makes up for the limitation that RGB-D cameras cannot be applied to endoscopes [22] and uses binocular endoscopes to achieve dense reconstruction and splicing of organs in the endoscopic scene. At the same time, the computational efficiency is higher than the monocular depth prediction results obtained through the deeper neural network [6,7], thus meeting the requirements of real-time reconstruction, which is of great significance for clinical application.

At present, our method is still limited to stomach models and pig stomachs in vitro, and further experiments have not been carried out in animals. At the same time, due to the difficulty of obtaining pathology-related data, our algorithm did not conduct more experiments on tissues containing lesions, which is also our next research direction. Because of the more complex structure of the human body, the effectiveness of this method in clinical practice has yet to be verified. In our research, only rigidity is considered at present. In subsequent studies, errors caused by non-rigid deformation caused by pneumoperitoneum [25,26], respiration, and other factors should be further considered so as to further improve the algorithm. With the gradual development of light source technology, lasers and other technologies [27] have also been introduced into endoscopes. Currently, our algorithm is only verified in the environment of white light, and more experiments need to be conducted in other light source conditions in the future to verify the effectiveness of the algorithm.

## 5. Conclusions

The proposed real-time dense reconstruction algorithm based on StereoNet and ORB-SLAM and using a binocular endoscope has been verified in a stomach model and a real pig stomach. The binocular method of dataset construction proposed in this paper provides a new idea for dataset production. At the same time, StereoNet and ORB-SLAM under RGB-D mode were adopted to improve the computational efficiency and the density of the point cloud with 834,650 points, basically meeting the requirements for real-time performance. In terms of accuracy, the RMSE increased by 40.96% compared with the traditional SGBM algorithm. The three-dimensional organ scene map obtained by our algorithm provides accurate and sufficient data for surgical navigation, realizing the measurement of lesion size, and helping to realize key steps such as path planning and target analysis in surgical navigation. In addition, 3D and multi-modal data registration can be realized by combining our method with fluorescence multi-modal data, thus providing doctors with more abundant information. The findings of this study are helpful to improve the prognosis of MIS and improve the level of medical care being provided.

## Data Availability

Not applicable.
